# A Case of Hydrophobia

**Published:** 1785

**Authors:** 


V. *A Caſe of Hydrophobia. Communicated in a Letter to* Samuel Foart Simmons, *M. D. F. R. S. by Mr.* John Haighton, *Surgeon.*

HAVING attended to the ſymptoms of hydrophobia in a caſe that lately occurred in Southwark, I take the liberty of preſenting it to you. It was my particular wiſh to have rendered it more intereſting by adding an account of the appearances upon diſſection; and with this view I took particular pains to procure leave to examine the body after death, but without effect. I lament this the more, as pathologiſts are not yet agreed upon the true ſeat of the diſeaſe. Some late diſſections ſeem to ſhow that the oeſophagus is the part principally affected; but when theſe are contraſted with the obſervations of others *, it will prove

* See Philoſ. Tranſ. Vol. XLVIII. art. 68.

that this, though a frequent, is not an invariable appearance.

With refpect to the other phænomena, there is fuch diverfity, that no one particular appearance feems common to every fubject. It will be unneceffary at prefent to notice in what this variety confifts, as it may be eafily collected from the writings of Morgagni, Bonetus, and others. If this hint, by operating as a ftimulus on the minds of others, fhould induce them to extend their inquiries to this part of pathology, my views in troubling you with this cafe will be amply accomplifhed.

### C A S E.

William Macey, of Crown Court, Kent Street, Southwark, aged five years, was bit in the right ear by a mad dog, on Thurfday, Auguft 4th, 1785. The wound, which was very flight, was, when I faw him, fo completely healed as fcarcely to leave the flighteft veftige of the bite. No other attention was paid to it than the application of a little cerate, nor were any prophylactics adminiftered.

On Thurfday, Auguft 24th, (three weeks after the bite) he complained of a pain in the part bitten; but, on examination, nothing could could be difcovered. A few hours after he expreffed a confiderable uneafinefs in the epigaftric region of the abdomen. He took food as ufual all that day, and flept a little at night.

Friday, Auguft 25th, the pain in the abdomen ftill continued, refpiration began to be laborious; he refufed all refrefhment, was very reftlefs, and paffed the night in a ftate of the moft perfect vigilance.

Saturday, Auguft 26th, about one o'clock, when I firft faw him, he was fitting upon a woman's knee until his bed could be made ready. His face was pale, his eyes feemed fixed in his head, and his countenance was ftrongly expreffive of horror. The pupil was for the moft part dilated, yet, when he was brought into a ftronger light than common, it was capable of a high degree of contraction. The neck of his body was moderate; his tongue moift, but covered with a white mucus; his pulfe one hundred and twenty in a minute. He was able to give anfwers to queftions with as much fatisfaction as could poffibly be expected from a child of his age. His breathing was laborious. On being offered fomething to drink, he ftarted at the fight of it as if it had been an inftrument of death; yet a little time after (while I was prefent) he called for drink, and took about a tea-cupful of milk with feeming avidity.

When the fpafms were very violent, for they were not always equally fo, he would cry out with a tone expreffive of horror, "I will not "have any thing to drink;" and this even at times when nothing was offered to him. He was for the moft part rational, yet his imagination fometimes appeared difturbed; for he would point towards the bottom of the bed, and ftart as if fomething frightened him, although no apparent object could be difcovered. His eyes, though for the moft part fixed, and his countenance, though generally expreffive of horror, would fometimes affume an appearance of circumfpection; and he would look around him as if he apprehended fomething from the byftanders.

A few minutes after he had drank the milk, fome of it regurgitated in a ftate of partial coagulation, and mixed with a fluid of a cineritious colour. Whenever he fpoke, it feemed to be with an effort, which was fometimes fo confiderable as to give the idea of a perfon labouring to fpeak under fuffocation.

There seemed to be no deficiency in the movement of his lips, yet there was something particular in his voice: he was sometimes able to pronounce many words together with a proper degree of force; but on a recurrence of the spasm, which manifested itself by a convulsive twitch at the time, there would instantly be such a defect of sound, that his voice became reduced almost to a whisper.

His respiration was frequent, irregular, and laborious; and, on turning aside the bed clothes, I perceived that the chest was dilated chiefly by the intercostal muscles: for the ribs were elevated and depressed in a considerable degree, without a proportional alteration of figure on the abdomen.

At three o'clock I saw him again, but perceived no material alteration in the symptoms, except in the degree of violence. The physician who attended him had ordered leeches to be applied to the epigastric region of the abdomen, a fomentation with poppies, and a blistering plaster.

At six o'clock, after having been violently convulsed, he died.

*Walworth,*

Sept. 12, 1785.

VI. *A Case*

